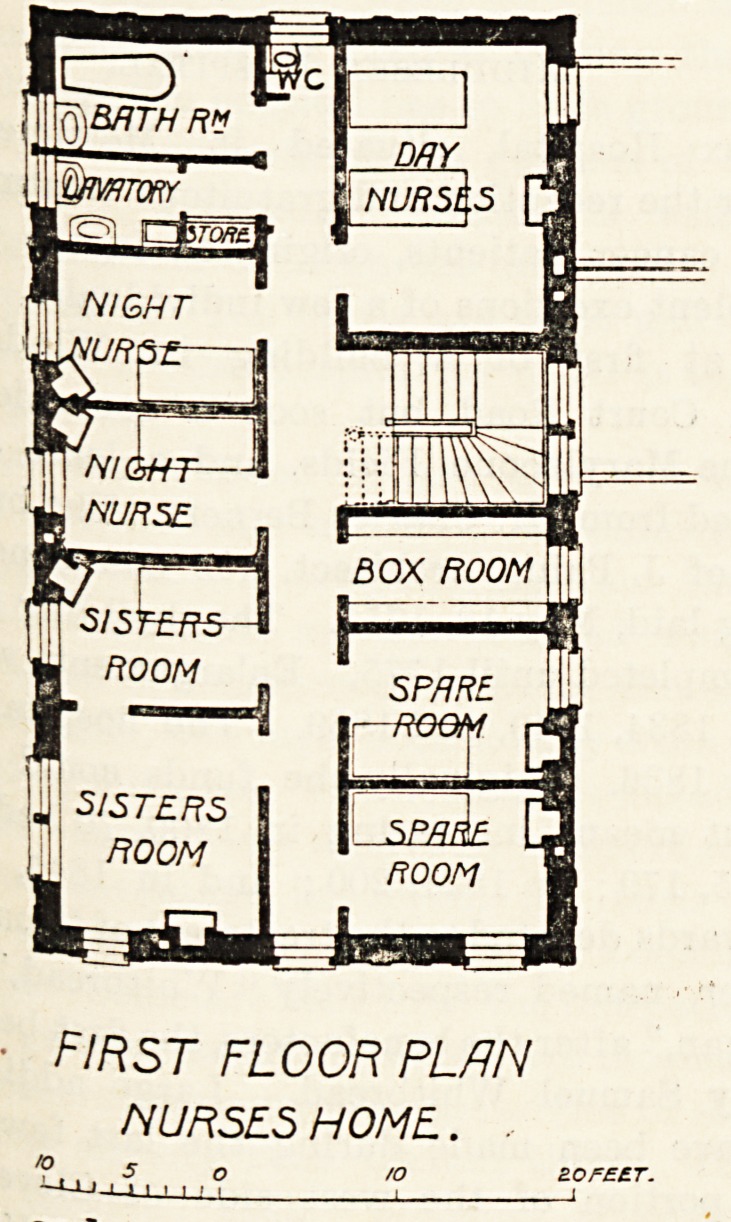# Lanfine Cottage Hospital for Incurable Cases of Consumption

**Published:** 1904-05-21

**Authors:** 


### LANFINE COTTAGE HOSPITAL FOR INCURABLE CASES OF CONSUMPTION.

THIS hospital has been erected in connection with the Broomhill Homes at Kirkintilloch, near Glasgow. These homes are for incurable diseases and this new hospital is intended for incurable cases of consumption only. It has been built about 300 feet west of the homes and is placed on ground which slopes towards the south. The hospital or ward part of the ground floor is arranged in broad-arrow form, which is a good arrangement for a hospital of this size and for this purpose. There are two wards, each for eight beds, one of these wards being for male patients and the other for women. The wards are well-proportioned and each bed has a window on either side. Glass-roofed verandahs run along the southern sides of these w[illegible] The sitting-room, which seems to be intended fo[illegible] sexes, is placed at the angle of union of the [illegible] and it, as well as the dormitories, has doors op[illegible] on to the verandahs. The passages leading to the [illegible] are lighted by skylights, and adjoining these pa[illegible] are the bath-room, closets and sinks. There are two sin[illegible] bedded wards placed alongside each other and fac[illegible] northwards. One of these has a window on its side as w[illegible] as at the end, and so has some cross-ventilation; but t[illegible] other has an end window only, hence such cross-ventila[illegible] as it may possess must be got by the open door, or by [illegible]

fanlight over the door, and even then it is only ventilation into a passage. The wards contain about ninety superficial

FIRST FLOOR PLAN
NURSES HOME.

10 5 0 10 20 FEET.

feet per bed, and assuming a ceiling height of 12 feet (it is probably more in this case as there are dormer windows shown in elevation) would give nearly 1,100 cubic feet per bed, which is sufficient for the purpose as the wards are well cross-ventilated. We feel bound to take exception to the position of the closets and sinks. These should certainly have been cut off from the main building by properly-ventilated passages. As to single-bedded wards, cross-ventilation is always more or less difficult, but something might have been done here by running them a a few feet further northwards. The hospital part of the building described above is connected to the Nurses' Home part by a corridor, from the south side of which the entrance porch projects, and on the opposite side is the laundry. The Nurses' Home is of two stories, and contains sufficient accommodation compactly and conveniently arranged.

The ward floors are of polished maple, and the walls are finished in Robinson's cement preparatory to being painted. The windows are correctly constructed in two sections, the lower part being a double casement, and the upper a hopper opening by a quadrant. The warming is also on the correct principle of open fireplaces supplemented by hot-water radiators under the windows. Fresh air is admitted there, and there are foul air extractors in the roofs. The outer walls are of brick with a hollow space, and they are covered with rough cast. The roofs are tiled. The cost was £4,000, which sum was generously given by Miss Brown, of Lanfine. The architects were Messrs. James Salmon and Son, of Glasgow.

## Figures and Tables

**Figure f1:**
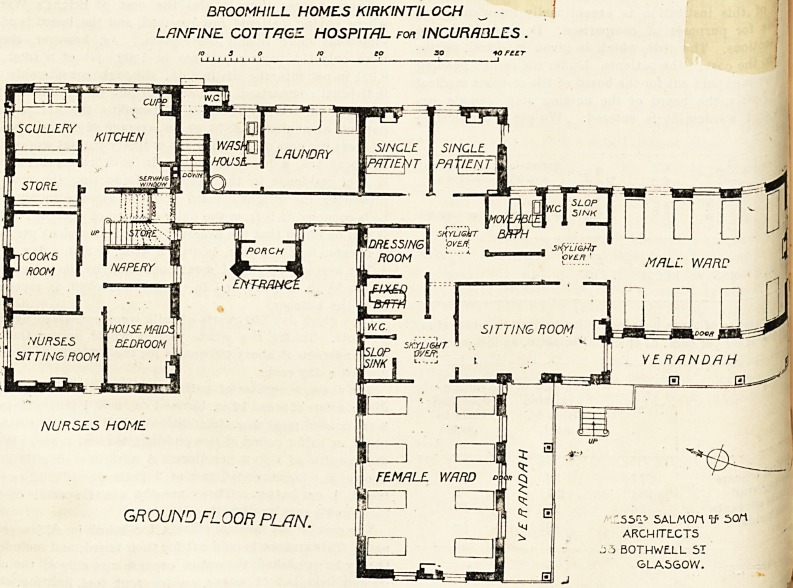


**Figure f2:**